# Differences in Parasite Infection Status and Behavior Between a Clonal Fish and Her Sexually Reproducing Heterospecifics

**DOI:** 10.1002/ece3.74288

**Published:** 2026-09-10

**Authors:** Kirsten Sheehy, Jonathan Aguiñaga, Mary Janecka, Jessica Stephenson, Kate L. Laskowski

**Affiliations:** ^1^ Department of Evolution & Ecology University of California Davis Davis California USA; ^2^ Powdermill Nature Reserve, Carnegie Museum of Natural History Rector Pennsylvania USA; ^3^ Department of Zoology Stockholm University Stockholm Sweden

**Keywords:** behavior, clonal, fish, parasites

## Abstract

Parasites impose strong selective pressures on animals, influencing traits from behavior to reproductive strategies. In particular, parasite‐mediated selection has been proposed as a major driver of sexual reproduction, as genetic recombination can generate variation that facilitates rapid evolutionary responses to parasites. Accordingly, asexual vertebrates are predicted to be more susceptible to parasites, yet some persist over longer than expected evolutionary timescales. The Amazon molly ( *Poecilia formosa* ) is a gynogenetic, clonal fish that occurs sympatrically with its sexual parental species, including the Sailfin molly ( *P. latipinna* ). Here we investigate the link between behavioral differences and parasite infection in wild‐caught Amazon and Sailfin mollies across two populations. Fish were exposed to a simulated predator attack, and space‐use behavior was quantified as time spent in open versus sheltered areas. We subsequently determined their infection status and parasite load via dissection. We found that Amazon and Sailfin mollies differed in their qualitative, but not quantitative resistance to parasites. Amazon mollies had worse qualitative resistance (i.e., were more likely to be infected with at least one parasite) and spent more time in the open, “riskier” portion of the arena. However, consistent with previous studies, Amazon and Sailfin mollies did not differ in quantitative resistance (i.e., parasite load). Parasite prevalence and load differed between our two collection sites, suggesting an important role for environmental context. These results suggest that there is a correlation between behavior and parasite exposure in Amazon mollies, while immune or physiological traits may allow them to limit parasite loads once infected. While the scope of this study makes it difficult to tell whether increased risk‐taking behavior leads to parasite exposure or vice versa, our results highlight important differences between sexual and asexual species in parasite‐laden environments.

## Graphical abstract

We found that Amazons and Sailfins differed in their qualitative, but not quantitative, resistance to parasites. Amazons had worse qualitative resistance (i.e., were more likely to be infected with at least one parasite) and spent more time in the open, riskier portion of the assay arena. Parasite prevalence and load differed between our two collection sites, suggesting an important role for environmental context.

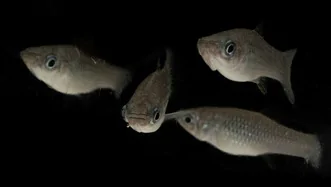

## 1 Introduction

Parasites can have profound negative effects on animals. They can kill their hosts directly (Sindermann 1987), or through the spread of secondary infections. They can reduce reproductive activity by affecting the energy available for reproduction or by making hosts less desirable partners (Clayton 1991). Parasites can also make animals more likely to be eaten—including by manipulating their hosts to behave in riskier ways (Hatcher et al. 2006; Poulin 2013).

As a result, many organisms invest considerable energy into avoiding and defending against parasites in their environments. While the immune system plays a critical role in helping defend against infection, behavioral traits can also help animals reduce encountering parasites (i.e., exposure) in the first place, and combat parasites once infected (DeSimone et al. 2018; Gibson and Amoroso 2022). Mechanisms that allow an animal to deal with parasites are often described as either qualitative or quantitative resistance. Qualitative resistance refers to mechanisms, behavioral or otherwise, that allow an animal to avoid an initial infection. This is typically quantified as a binary response: did the animal become infected at all? Quantitative resistance refers to mechanisms that allow an animal to minimize the quantity of parasites they are host to once infected, typically measured using parasite load. There is no single strategy for success. For example, sleepy lizards (*Tiliqua rugosa*) that used more refuge spots had lower tick loads than those that used fewer, shared refuges (Leu et al. 2010), whereas chipmunks ( *Tamias sibiricus* ) with higher space use had higher tick loads (Boyer et al. 2010). Similarly, animals can adopt different strategies to manage parasites once infected. For example, fruit flies decrease their feeding rates, increasing the amount of time they can survive with *Salmonella typhimurium* infections (Ayres and Schneider 2009). Meanwhile, crickets ( *Gryllus texensis* ) respond to infection by changing their foraging—they prioritize a diet lower in fat which supports better immune function (Adamo et al. 2010). There are thus a variety of behavioral responses animals use to limit parasite exposure and improve their defenses when infections occur.

As host behavior may alter exposure to parasites, parasites can in turn directly and indirectly influence their hosts' behavior. Parasites can directly influence host behavior in an attempt to increase transmission or for reproductive purposes (Poulin 2013). For parasites with a multi‐host lifecycle, behavioral manipulation can be an attempt to get their current host eaten by their terminal host. For example, stickleback ( *Gasterosteus aculeatus* ) infected with cestodes ( *Schistocephalus solidus* ) reduce wall‐hugging behavior, a space‐use pattern that is frequently considered to be an anxiety or anti‐predator related behavior (Colwill and Creton 2011; Grécias et al. 2018; Schnörr et al. 2012; Treit and Fundytus 1988). Parasites can also incidentally affect host behavior by increasing metabolic costs or restricting host movement, such as the case of ectoparasites increasing drag on aquatic creatures (Hockley et al. 2014; Poulin 2013). For example, guppies ( *Poecilia reticulata* ) infected with more ectoparasites (*Gyrodactylus*) had reduced rheotaxis and spent more time in low‐flow areas (Blondel et al. 2024). Examining relationships between individual behavior and parasite infection can reveal different strategies used by animals in response to parasites.

Given how much influence parasites can have on animals, this is one reason why we think sexual reproduction evolved in the first place (Hamilton et al. 1990). Sexual recombination, along with genetic recombination, can produce more variation at loci related to parasite resistance (Bremermann 1980; Hamilton 1980; Jaenike 2005; Levin 1975). This variation can then be selected upon, allowing species to evolve in response to parasite pressures. Indeed, there is good evidence for parasites inducing strong rapid selection on many traits among animals. For example, allele frequencies in the major histocompatibility complex of stickleback shifted adaptively over the course of two generations in response to different parasites (Eizaguirre et al. 2012). Without sexual recombination, asexual species are expected to have less variation at loci related to parasite resistance, and thus be unable to quickly evolve responses to parasites (Bell 2019; Valen 2014). A key prediction is that asexual species may be more susceptible to parasite infection in the first place (i.e., worse qualitative resistance) and also may be less well equipped to manage infections when they do happen (i.e., worse quantitative resistance).

**FIGURE 1:**
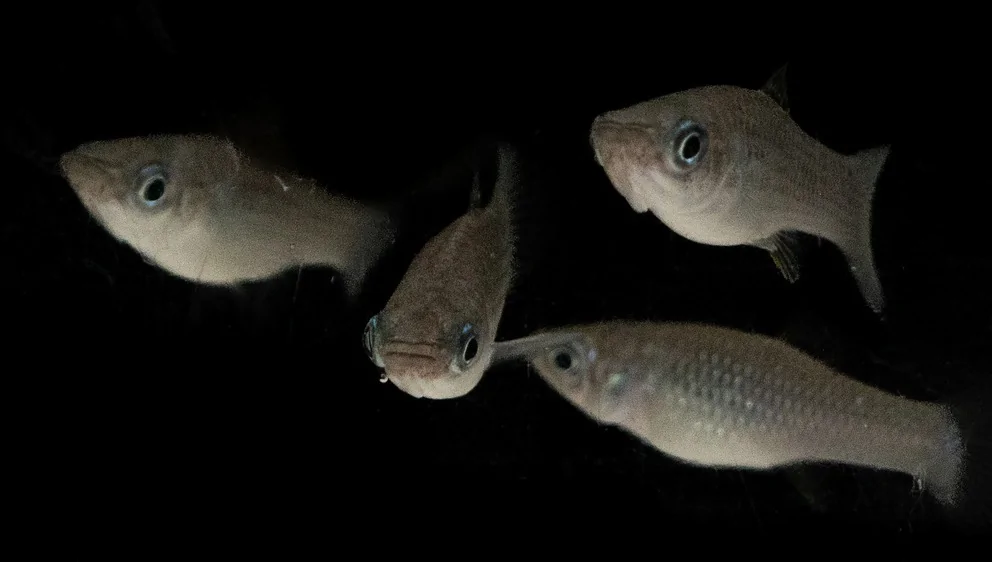
The Amazon molly ( *P. formosa* ) is a small, gynogenetic species of fish that lives in brackish water along the Gulf of Mexico. She is all female and effectively a clone, producing offspring with perfect diploid copies of her genome. Photograph by Dr. Jonathan Aguiñaga.

This may especially be the case for larger (i.e., multicellular) asexual species which have significantly longer lifespans than the parasites themselves, such as unisexual vertebrates. But, despite these intense pressures from parasites, asexual vertebrates do persist over meaningful timescales (Avise 2015; Lampert and Schartl 2008). For example, the Amazon molly ( *Poecilia formosa* ), is a small gynogenetic species of fish that lives in brackish water along the Gulf of Mexico (Schlupp et al. 2002) (Figure 1). Effectively a clone, she produces eggs which contain perfect diploid copies of her genome. She therefore has relatively little among‐individual genetic variation. However, she does maintain high within‐individual variation via high heterozygosity—likely due to her origin, a hybridization event approximately 100,000 years ago between the Sailfin molly ( *P. latipinna* ), and the Atlantic molly ( *P. mexicana* ) (Lampert and Schartl 2008; Schartl et al. 1995). Additionally, the Amazon molly is an obligate sperm parasite; she must mate with males from either of her parental species to stimulate embryogenesis but this paternal DNA is excluded from the developing embryo (Schlupp et al. 2002). Because the Amazon molly still requires sperm to reproduce, she must be sympatric with at least one parental species. Thus, the asexual Amazon molly and her closely related parental species, the Sailfin molly, share otherwise similar ecologies and habitats. This provides an excellent opportunity to examine how the Amazon molly has persisted alongside her sexually reproducing parental species, especially in the face of parasites. There have been a few previous studies that have compared quantitative resistance between the Amazon molly and her sexual parental species and found no differences in overall parasite loads (Tobler and Schlupp 2005; Gösser et al. 2019); however, given spatial and temporal variation in parasite communities (Poulin 2020; Tobler and Schlupp 2005), it is not clear how general or stable this pattern may be.

To explore the relationship between behavior and parasites in this system, we collected Amazon and Sailfin mollies from the wild and measured their behavior in response to a simulated predator attack. We then quantified each individual's gill parasite load to test for a relationship between behavior and parasite load and whether this relationship differed between the two species. We focus on gill parasites because they are known to impact ecologically relevant behaviors such as swimming speed and predator responsiveness and are more likely to be preserved after collection, enabling better identification (Crane et al. 2011; Santos and Santos 2013).

## 2 Methods

### 2.1 Collection

We collected Amazon and Sailfin mollies from two sites in southern Texas, between August 7 and 12, 2022. In total, we collected 69 Amazon and 59 Sailfin mollies from two sites. We collected fish using a combination of seine and dip nets. We then transported fish in 19 L buckets filled with water from their site back to our temporary arena setup. A subset of the fish was immediately euthanized by rapid decapitation and placed in vials of ethanol for future parasite dissections. All protocols are approved within UC Davis IACUC protocol #21897. Collection permit SPR‐0722‐099 was issued by Texas Parks and Wildlife.

### 2.2 Husbandry

To keep track of individuals and to minimize handling, we placed each fish in a labeled, transparent plastic cup with holes punched in the cup. Each cup was approximately 150 mL in volume. These cups were then placed in a larger, water‐filled 19 L bucket with an air stone, which kept the water aerated and gently moved the plastic cups within the bucket. Fish were housed in these individually labeled cups and buckets whenever they were not being assayed. Before being placed in cups, we photographed each fish against a ruler and extracted length measurements from the photos using QuPath (Bankhead et al. 2017). We started behavioral assays the morning after collection. After completing two behavioral trials, the fish were euthanized and placed in vials of alcohol for parasite dissections.

### 2.3 Behavioral Assays

**FIGURE 2:**
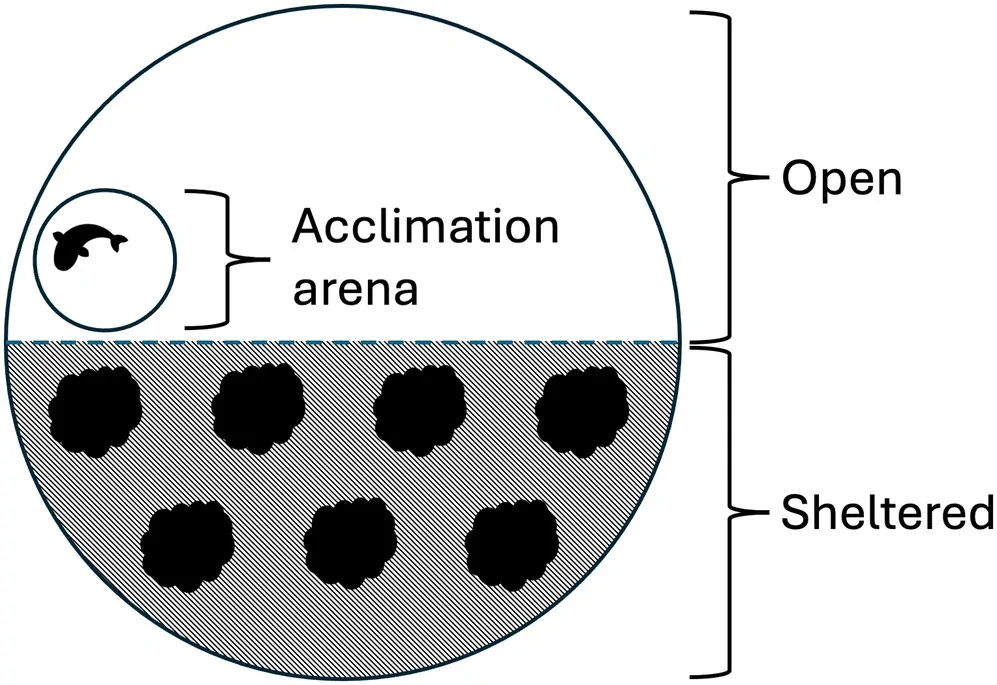
Behavioral assay arena. The sheltered half of the arena had seven black trash bag “plants” that served as refuges. Fish were placed into the smaller, acclimation tub for 10 min, before being gently poured into the middle of the arena. We then measured the amount of time spent in the sheltered (black striped) half of the arena or the open half (white).

We assayed fish in a 1.5 m diameter arena filled to a 6 cm depth with water from their collection site. One half of the arena had artificial plants made from black trash bags and served as a refuge. The other half of the arena contained no artificial plants and was considered an open, potentially “risky” area (Figure 2). We consider the open half of the arena to be risky because open water often presents a higher risk of predation for this small fish. We removed a fish from its individual cup and placed it in a 15 cm diameter acclimation tub placed in the open half of the arena. After 10 min in the acclimation tub, we gently poured the fish into the larger arena and the acclimation tub was removed. After allowing the animal to explore the arena undisturbed for 10 min, we simulated a predator striking the water by slapping the surface of the water with a foam pool noodle. After 10 more minutes, the trial concluded and we returned fish to their labeled cup in an aerated bucket. Fish repeated this assay two times in 1 day, with at least 1 h between trials. Fish were subsequently euthanized and preserved in alcohol for future parasite analyses. We extracted behavioral metrics from blinded video recordings of the trial using BORIS (Friard and Gamba 2016). All observations were rounded to whole seconds. For this analysis, we only looked at time points after the startle stimulus. We recorded the amount of time in the open or sheltered halves of the arena and the number of times the animal switched between the open and sheltered portions of the arena as our behavioral metrics of interest.

### 2.4 Parasite Dissections

From January through April 2023, we dissected the preserved mollies ( *P. formosa* and *P. latipinna* ) in the laboratory to obtain estimates of parasite infection intensity and prevalence. Complete dissections were first carried out for five specimens. Based on these dissections, we determined that the decomposition of internal parasites in larger fish made these specimens unsuitable for a complete assessment of the parasite community. Parasites encysted in or attached to the gills could be reliably counted regardless of host size or level of decomposition. Gill tissue from the preserved specimen was separated under a dissecting scope. We counted the total number of parasites encysted in or attached to the gill filaments on each gill. Most infections consisted of trematode metacercaria from the families Echinochasmidae and Heterophyidae. The identification was conducted by an experienced observer (M.J.), based on the morphology of the parasites, the tissue they were infecting, and the geographic location from which they were collected. Trematodes in these families exhibit complex life cycles completed by sequentially infecting operculate snails, fish, and aquatic birds (Yamaguti 1975). The gills of several specimens were also infected with unidentified parasitic copepods. These were relatively uncommon (505 out of 5465 gill parasites observed, or approximately 9%) and occurred at similar relative abundances across sites and species. At Weslaco, 13.2% of parasites were copepods in Sailfin mollies and 13.4% in Amazon mollies. We have a larger difference between species at Brownsville: 9% copepods in Sailfin mollies and 14.9% in Amazon mollies. Fish from Brownsville generally had far more parasites (both trematodes and copepods), so more variation is unsurprising. The total parasite count was almost perfectly correlated with the count of trematodes alone (*r =* 0.998), so we elected to include unidentified copepods in the total count of gill parasites for all analyses.

### 2.5 Statistical Analyses

Our analysis sought to answer two main questions: Are there differences in behavior and/or parasite load between Amazon and Sailfin mollies? And is there a relationship between parasite load and behavior?

First, we tested whether parasite load varied between Amazon and Sailfin mollies. Initial testing revealed that the parasite data were zero inflated. Our smaller sample size (69 Amazon mollies and 59 Sailfin mollies) limited our ability to fit more complex hurdle models. Instead, we fit two generalized linear mixed models: a count model to assess the total number of parasites assuming a negative binomial distribution (i.e., parasite load) and a logistic model to assess the binary presence or absence of parasites (i.e., infection status). General linear mixed models are well suited to our data, as they are robust to uneven sample sizes (Pinheiro 2014). In the count model, the data was filtered to only include parasite counts larger than zero. The response variable was the total number of parasites and the predictor variables initially included were sex, standard length, species ( *P. formosa* or *P. latipinna* ), site (Weslaco and Brownsville), and the interaction between species and site. We used the same predictor variables for the logistic model, but the response variable was the binary infection status—with or without parasites. For both models, we performed backwards model selection to arrive at the simplest model. We used log likelihood ratio tests to test the effect of sex and the interaction between species and site; however, neither effect was significant. So, to increase power in our small sample size, we removed these effects and report the model with only the main effects of species, site, and standard length (Kuznetsova et al. 2017). We evaluated model assumptions with diagnostic plots (Hartig 2024). When effects were statistically significant, we estimated effect sizes using a log likelihood ratio test (Zeileis and Hothorn 2002).

**FIGURE 3:**
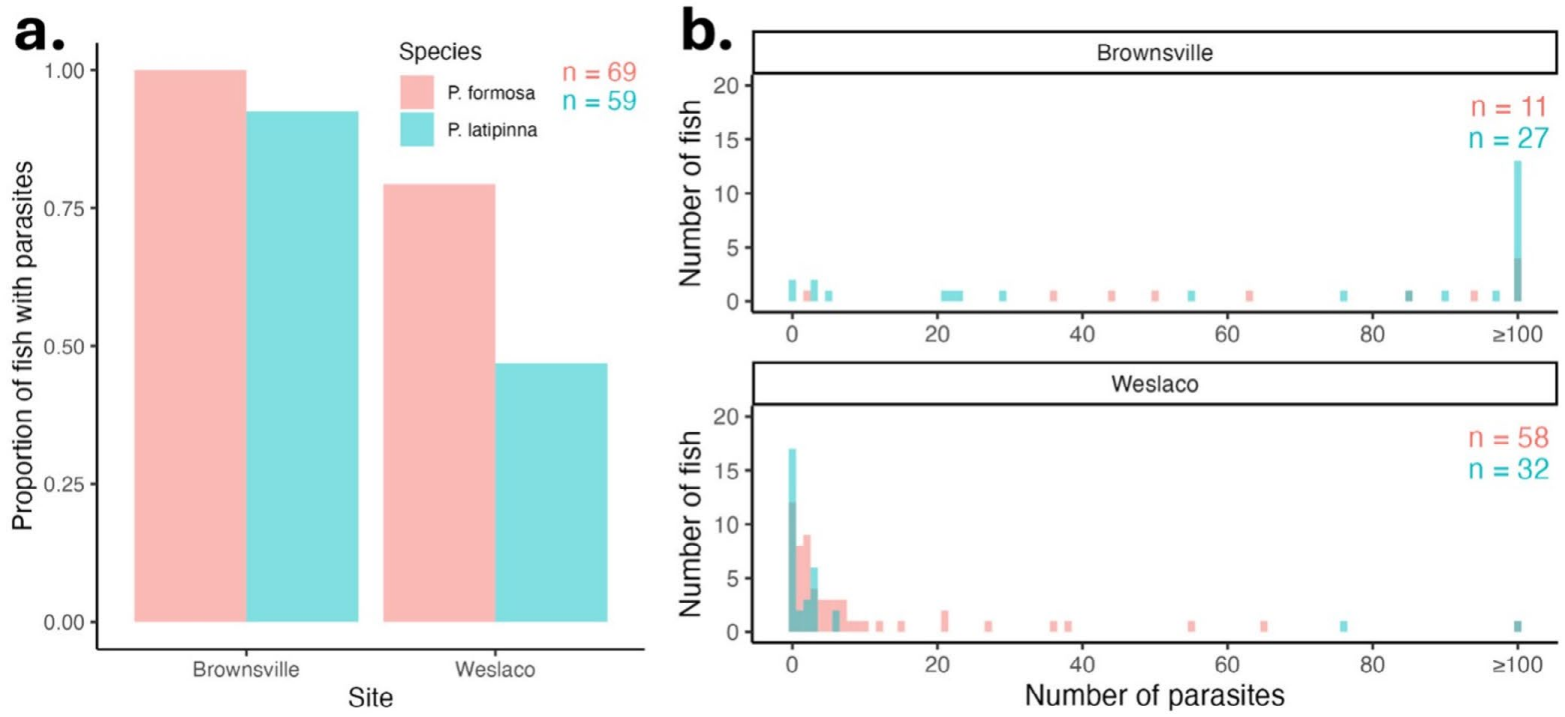
Parasite counts by species. Amazon mollies ( *P. formosa* ) are pink and Sailfin mollies ( *P. latipinna* ) are blue. (a) Most fish of both species have at least one parasite at the Brownsville site. There are fewer fish infected with parasites at the Weslaco site, and there is a more pronounced difference between the two species. (b) Site was a significant predictor of parasite load, with fish from Brownsville having more parasites. Conversely, fish from Weslaco were more likely to have no parasites at all.

Given that the two sites differed in overall parasite loads (Figure 3), we also analyzed the two sites separately, repeating the analysis described above. For each site, the models only included the effect of species and standard length.

Second, we examined whether parasite load affected the behavior of the mollies and whether that relationship differed between species or site (20 Amazon mollies and 41 Sailfin mollies). We ran models for each behavioral metric: time spent in the open half of the arena after the startle and whether fish entered the open half of the arena at all. Both response variables were also zero inflated because many fish spent the entire trial hiding in the covered half of the arena (i.e., spent zero time in the open). We fit two generalized linear mixed models: a count model to assess the total amount of time spent in the open (i.e., only with non‐zero data points) and a logistic model to assess the binary response of leaving the sheltered half or not. We initially included the following predictors in both the negative binomial and logistic models: sex, total number of parasites, species ( *P. formosa* or *P. latipinna* ), site (Brownsville or Weslaco), as well as body size (standard length in centimeters). We initially tested for interactions among these terms using a log likelihood ratio test; however, these interactions were not significant, so they were dropped from the model that we report in the main results (Kuznetsova et al. 2017). Similarly, sex and standard length did not significantly improve model fit per log likelihood tests and were dropped from the final model.

To confirm that patterns were not being driven by site level differences in overall parasite load, we also repeated this analysis for the two sites separately. The initial model and model selection process was the same, except that the variable “site” was dropped from the initial model.

All analyses were conducted using R version 4.3.1 (R Core Team 2023).

## 3 Results

### 3.1 Parasites

We found some evidence that the sexual Sailfin molly and asexual Amazon molly differed in infection status (Table 1, Figure 3). The effect of species on the presence or absence of parasites was significant for the binomial model (LLR = −53.89, *p* = 0.001) (Table 1). This indicates that Sailfins are 15.79% more likely to not be infected (i.e., have no parasites), when compared to Amazon mollies (Figure 3c). There was no effect of species on the total number of parasites among infected fish, as shown in the count model (LLR = −377.71, *p* = 0.838). Additionally, we found that there were strong site‐level differences, with fish from Weslaco both being less likely to be infected (LLR = −63.738, *p* = 0.0005; 5.40% less likely to be infected) and having fewer parasites overall when they were infected (LLR = −412.45, *p* = 2e‐16; 90.67% fewer parasites; Figure 3c). Additionally, standard length was a significant predictor of how many parasites a fish was likely to have (LLR = −391.49, *p* = 5.12e‐9); an increase in length by 1 cm led to 161.40% more parasites.

**TABLE 1:** Results from the generalized linear mixed models for count and binary data examining whether species or site affects the number or presence of parasites.

Parasite models			
Effect	Estimate (SE)	LLR	*p*
Count model: number of parasites ~ species + site + standard length			
Species: Sailfin	−0.053 (0.260)	−377.71	0.838
Site: Weslaco	−2.644 (0.264)	−412.45	2e‐16
Standard length	0.961 (0.164)	−391.49	5.12e‐9
Binomial model: infection status ~ species + site + standard length			
Species: Sailfin	−1.674 (0.513)	−53.89	0.001
Site: Weslaco	−2.864 (0.821)	−63.738	0.0005
Standard length	0.253 (0.340)	−54.169	0.457

*Note:* Initial models included sex and the interaction between species and site, but these terms were dropped during the model selection process. We only report the final, reduced models in this table.

Given the difference in overall parasite load between the two sites, we repeated this analysis for each site separately. The independent site analysis from Weslaco also found that Sailfins were less likely to be infected than Amazon mollies, but we did not see any evidence for species level differences at the Brownsville site. At both sites, longer fish had more parasites (Tables 2 and 3). At the Weslaco site, species had no effect on the total number of parasites (LLR = −181.81, *p* = 0.450) but did affect the infection status of fish (LLR = −51.952, *p* = 0.002), with Sailfins being 16.23% more likely to have no parasites. Length did contribute to overall parasite load, with longer fish having 209.26% more parasites per centimeter (LLR = −190.56, *p* = 5.72e‐6). For the Brownsville site, species had no effect on the infection status or parasite load of a fish (Table 3). Length contributed to overall parasite load, with longer fish having 113.4% more parasites per centimeter (LLR = −199.72, *p* = 1.73e‐4).

**TABLE 2:** Results from the generalized linear mixed models for count and binary data examining whether species affects the number or presence of parasites at the Weslaco site.

Weslaco parasite models			
Effect	Estimate (SE)	LLR	*p*
Count model: number of parasites ~ species + standard length			
Species: Sailfin	−0.288 (0.381)	−181.81	0.450
Standard length	1.129 (0.249)	−190.56	5.72e‐6
Binomial model: infection status ~ species + standard length			
Species: Sailfin	−1.641 (0.524)	−51.952	0.002
Standard length	0.281 (0.355)	−47.012	0.428

*Note:* We used the same model structure found in Table 1, but do not include site.

**TABLE 3:** Results from the generalized linear mixed models for count and binary data examining whether species affects the number or presence of parasites at the Brownsville site.

Brownsville parasite models			
Effect	Estimate (SE)	LLR	*p*
Count model: number of parasites ~ species + standard length			
Species: Sailfin	0.165 (0.331)	−194.10	0.618
Standard length	0.758 (0.202)	−199.72	1.73e‐4
Binomial model: infection status ~ species + standard length			
Species: Sailfin	−17.123 (3240.14)	−7.720	0.996
Standard length	−0.108 (1.135)	−6.970	0.924

*Note:* We used the same model structure found in Table 1, but do not include site.

### 3.2 Behavior

**FIGURE 4:**
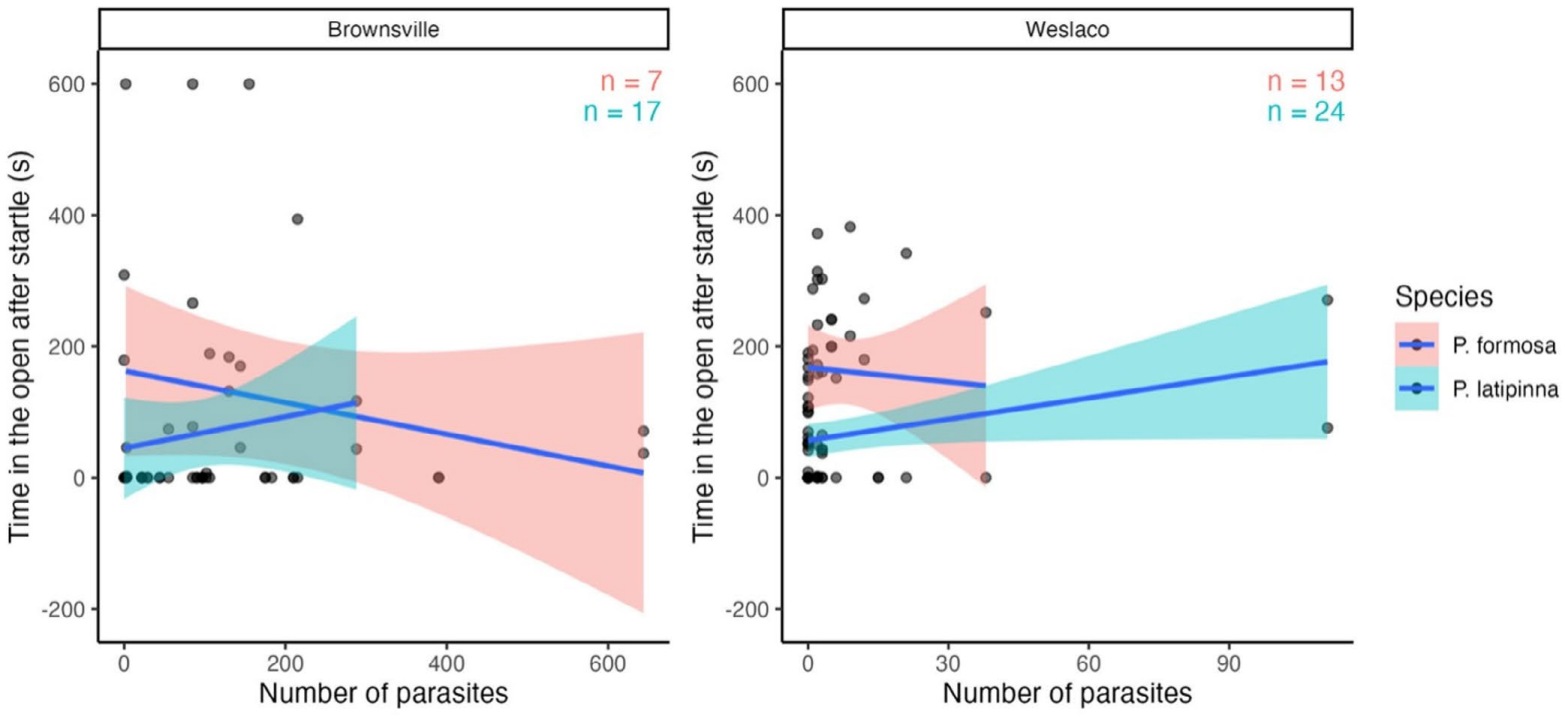
Amount of time in the open versus the number of parasites for each species. Data is split by site. The total number of parasites had a significant effect on the amount of time that fish spent in the open, but the effect size was quite small—0.20% less time in the open for every additional parasite. There was no interaction between species and the number of parasites.

**FIGURE 5:**
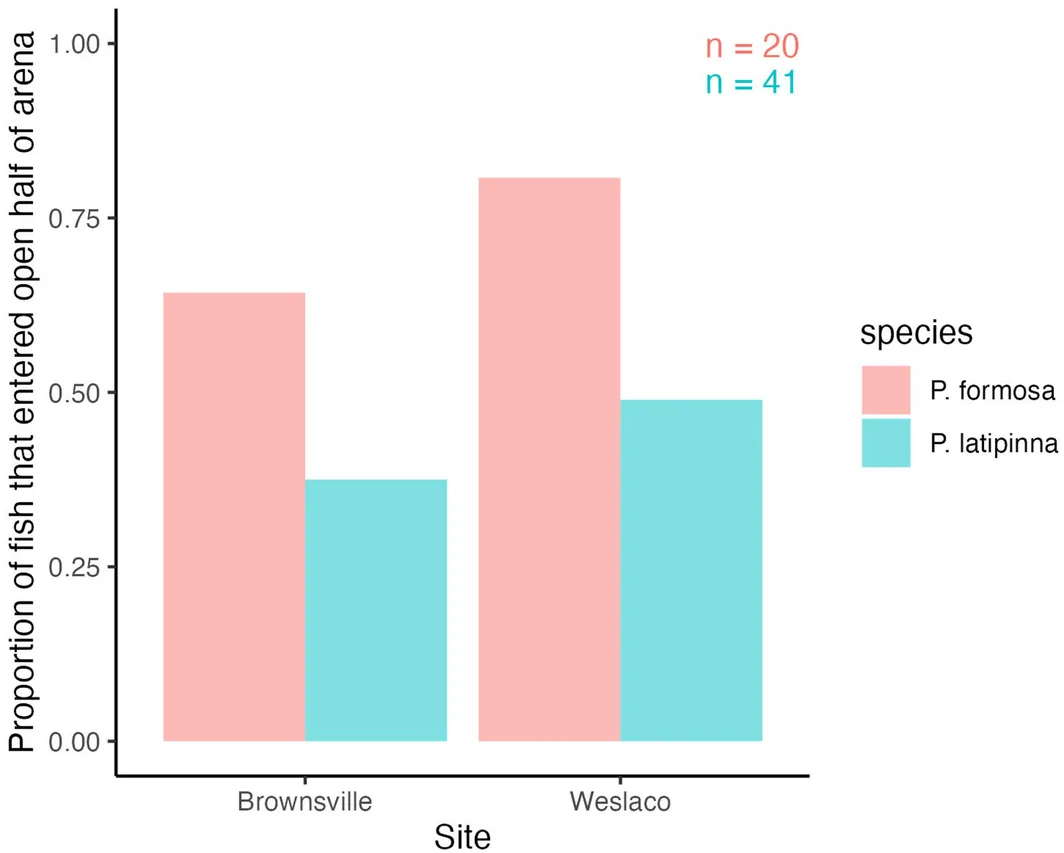
Proportion of fish that entered the open half of the arena at least once after the startle stimulus. Amazon mollies were more likely to spend at least some proportion of the trial in the open.

We found that how fish responded to the simulated predator attack was predicted by the total number of parasites and species (Table 4). The total number of parasites was a significant predictor for the count model (LLR = −397.32, *p* = 0.048) though this effect was quite small: for each additional parasite, there was a 0.20% decrease in the amount of time spent in the open—about 1 s less over a 10‐min period (Figure 4). The effect of species on fish entering the open half of the arena was significant for the binomial model (LLR = −79.88, *p* = 0.004) indicating that Sailfin mollies are 22.01% less likely to spend any time in the open when compared to Amazon mollies (Figure 5).

**TABLE 4:** Results from the generalized linear mixed models for count and binary data testing the effect of number of parasites, species, and site on whether a fish spends any time in the open half of the arena and how much.

Behavior models			
Effect	Estimate (SE)	LLR	*p*
Count model: time in open after startle ~ species + site + total number of parasites			
Species: Sailfin	−0.229 (0.213)	−396.66	0.281
Site: Weslaco	−0.496 (0.272)	−397.71	0.068
Total number of parasites	−0.002 (0.001)	−397.32	0.048
Binomial model: enters open half of arena at least once ~ species + site + total number of parasites			
Species: Sailfin	−1.260 (0.440)	−79.88	0.004
Site: Weslaco	0.760 (0.483)	−76.71	0.116
Total number of parasites	0.002 (0.002)	−75.683	0.509

*Note:* Initial models included sex, standard length, and the interaction between species and site, but these terms were dropped during the model selection process. We only report the final, reduced models in this table.

Similar to the parasite analysis, we also investigated each site separately and found similar results to the combined site analysis. There were significant effects of species and total number of parasites on behavior in Weslaco, but not Brownsville (Tables 5 and 6). Sailfin mollies from Weslaco spend 35.27% less time in the open than Amazon mollies (Table 5). Similarly, Sailfin mollies from Weslaco were 6.32% more likely to spend the entire trial in the sheltered half (Table 5). Additionally, fish from Weslaco with more parasites were 47.7% more likely to spend the entire trial in the sheltered half of the arena, though these results were marginally significant (LLR = −47.682, *p* = 0.054) (Table 5). In Brownsville, species and the total number of parasites had no effect on the amount of time fish spend in the open, nor whether they entered the open half of the arena (Table 6).

**TABLE 5:** Results from the two generalized linear mixed models for count and binary data examining whether species and total number of parasites affects the amount of time that fish from Weslaco spend in the open.

Weslaco behavior models			
Effect	Estimate (SE)	LLR	*p*
Count model: time in open after startle ~ species + total number of parasites			
Species: Sailfin	−0.435 (0.220)	−263.7	0.048
Total number of parasites	−0.005 (0.005)	−262.3	0.315
Binomial model: enters open half of arena at least once ~ species * total number of parasites			
Species: Sailfin	−2.696 (0.883)	−50.854	0.002
Total number of parasites	−0.092 (0.048)	−47.682	0.054
Species: Total number of parasites	0.217 (0.201)	−47.278	0.281

**TABLE 6:** Results from the two generalized linear mixed models for count and binary data examining whether species and total number of parasites affects the amount of time that fish from Brownsville spend in the open.

Brownsville behavior models			
Effect	Estimate (SE)	LLR	*p*
Count model: time in open after startle ~ species + total number of parasites			
Species: Sailfin	0.149 (0.464)	−130.90	0.748
Total number of parasites	−0.002 (0.001)	−131.91	0.094
Binomial model: enters open half of arena at least once ~ species + total number of parasites			
Species: Sailfin	−0.953 (0.669)	−31.027	0.173
Total number of parasites	0.002 (0.002)	−30.295	0.519

## 4 Discussion

Our study adds to previous work documenting and comparing the presence and quantity of parasites between the asexual Amazon molly and one of her sexual parental species, the Sailfin molly. Among infected fish, there was no difference in the quantity of gill parasites between the two species. This is in line with previous work, which also found that there was no difference in the parasite loads of Amazon and Sailfin mollies (Tobler and Schlupp 2005). However, in our study, we found that Amazon mollies were more likely, compared to the Sailfin mollies, to be infected with at least one gill parasite (Figure 3a). Additionally, we observed their space use behavior and found that Amazon mollies are more likely to spend time in the open, “riskier” half of the arena than Sailfin mollies (Figures 2 and 5).

So why might the Amazon molly be more likely to be infected, but carry similar numbers of gill parasites to infected Sailfin mollies? As previously mentioned, this is not the first time that similar parasite loads, and thus similar quantitative resistance, between these two species have been observed (Tobler and Schlupp 2005). Indeed, the Amazon molly's evolutionary longevity as a clone and her coexistence with parasites has been a topic of interest for some time. But resistance to parasite infection is complicated. One possible explanation for these similarities in quantitative resistance is that the Amazon molly has higher heterozygosity than their sexually reproducing partners (Warren et al. 2018). Additionally, immune genes in the Amazon molly are equally diverse in the Amazon and Sailfin mollies (Gösser et al. 2019; Schaschl et al. 2008; Warren et al. 2018). These patterns may be explained by the Amazon molly's hybrid origin; with an Atlantic molly mother and a Sailfin molly father, she may have inherited more heterozygous and diverse immune genes than either of her parental species (Schartl et al. 1995). And so once infected, Amazon mollies may be as capable of fighting continued infection as her sexual parental species. However, these patterns do not explain our results showing that the Amazon molly shows lower qualitative parasite resistance (i.e., is more likely to be infected at all).

**FIGURE 6:**
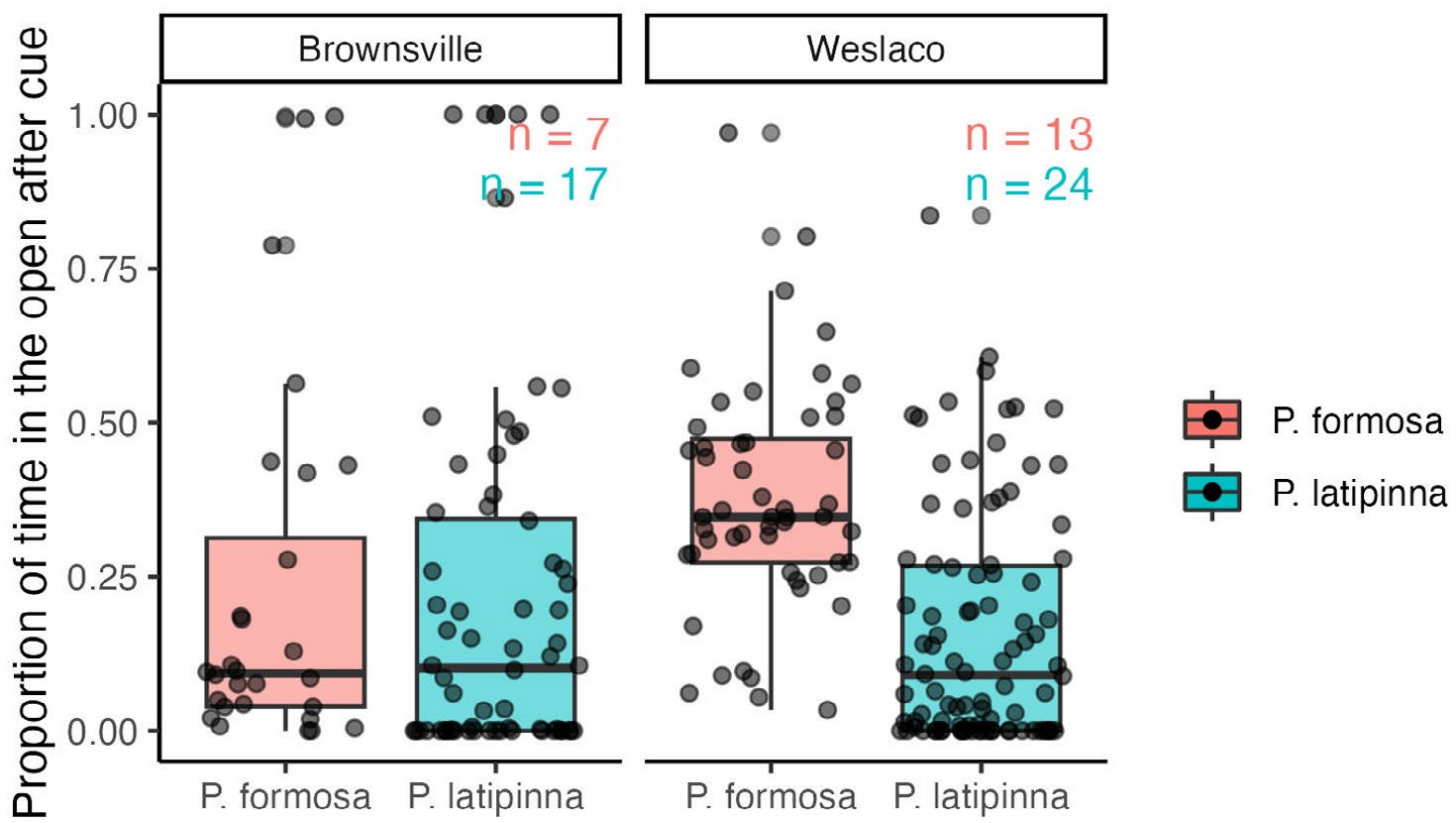
Proportion of time spent in the open after the startle stimulus. Data is split by site. There was no effect of species or site on the amount of time that fish spent in the open.

Perhaps these differences in qualitative resistance can be attributed to differences in behavior. Indeed, we found that Amazon mollies were more likely to spend at least some portion of the trial in the open, potentially “risky” part of the arena regardless of parasite load (Figures 6 and 5). This is also consistent with previous work, which has found that Amazon mollies are more active when compared to their other parental species, the Atlantic molly ( *Poecilia mexicana* ) (Gallagher et al. in review). Amazon mollies' tendency to enter the open half of the arena suggests that they may be more exploratory or risk taking, a behavior that could contribute to higher parasite encounter rates (i.e., exposure) simply by virtue of them using more of the available environment. These higher activity levels could affect their qualitative resistance, making them more likely to encounter parasites while their immune genes keep their quantitative resistance similar to the Sailfin mollies. It is important to note that while we observed these species‐level differences in infection status (i.e., qualitative resistance) and space use, there did not appear to be a strong relationship between parasite load (i.e., quantitative resistance) and behavior. Parasite load was a predictor of space use, but the effect size was so small that it is unlikely to have a large biological impact. For each additional parasite, there was a 0.20% decrease in the amount of time spent in the open—this works out to about 1 s less in the open over a 10 min period (Table 4).

Our study was correlational and therefore we are not able to test potential mechanistic links between behavior and parasite load. It is possible that parasites are directly or indirectly responsible for the behavioral differences we saw. Parasites may alter their hosts' behavior in a bid to increase transmission to their final host. For example, the acanthocephalan parasite, *Pomphorhynchus laevis*, increases risky behavior in the *Gammarus pulex* amphipod (Kaldonski et al. 2007). Parasites themselves also show considerable variation in their space use behavior, which could affect host avoidance behavior. Echinochasmidae are often positively phototaxic and actively swim in the water column while *C. fomosanus* are negatively phototaxic and found lower in the water column. Parasites also vary considerably in their infection modes. Endoparasites vary dramatically in their effects on hosts, from being mostly benign to causing necrosis (Feist and Longshaw 2008). Depending on the endoparasites present at our sites, we may have drawn different conclusions. For example, if ectoparasite and endoparasite loads are not correlated and ectoparasites reflect a smaller proportion of the infections we may have observed a stronger relationship between parasite load and the proportion of time spent in the open. Conversely, if endoparasites at our sites were mostly benign, then counting them may explain why (or eliminate) the weak relationship between parasite load and time in the open. We hope that further investigations into Amazon and Sailfin mollies, and their parasites are able to capture more causality in their relationships.

It is also worth noting the large environmental differences we saw between the two sites, which may be driving the variation in parasite loads we saw. This may be a function of two non‐mutually exclusive processes: (1) differences in flow rates and (2) differences in site conditions that favor the trematode's first intermediate host, snails. The Weslaco site was a channelized stream feeding into a larger resaca in Weslaco, Texas, with relatively high flow rates, high turbidity from upstream construction, and greater channel depth. Resacas are old oxbows that have been cut off from a river. The Brownsville site was a much smaller resaca under a freeway overpass in Brownsville, Texas. At the time of collection, there had been no significant recent rain, so the fish were in disconnected pools of shallow water of varying sizes. Characteristics of water bodies can significantly alter parasite abundance by diluting cercariae in three‐dimensional space (De Montaudouin and Lanceleur 2011). Increased stream depth is also associated with lower trematode density (Falke and Preston 2022). Reduced flow has been consistently associated with increased prevalence and intensity in parasites that use free‐living stages for transmission, including copepods and trematodes (Barker and Cone 2000; Medeiros and Maltchik 1999). The faster flowing water at the Weslaco site may have reduced the prevalence of parasites site‐wide or their ability to find hosts, allowing us to see species‐level differences in susceptibility to parasite infection. Alternatively, flow or other site differences may have altered patterns of parasite‐induced mortality, resulting in our observed differences in parasite load (Clark et al. 2024). While we did not quantify snail density, vegetation in and around the ponds was high at the Brownsville site and limited at the Weslaco site. High vegetation, combined with low flow rates and shallow water depth, is likely facilitating greater abundance of snail first‐intermediate hosts and increased trematode infection (Falke and Preston 2022). First intermediate host density can be a major driver of infection in subsequent hosts (De Montaudouin and Lanceleur 2011; Hechinger and Lafferty 2005). An abundance of parasites at the Brownsville site may have simply overwhelmed both species, resulting in no differences in infection rates or total parasite counts observed.

This study's results are consistent with previously observed patterns in the Amazon molly's space use (Gallagher et al. 2025) and parasite loads (Tobler and Schlupp 2005). We found that Amazon mollies are more likely to use open spaces than Sailfin mollies. It is possible that this behavioral difference we observed contributes to parasite exposure; the more exploratory Amazon molly may be more likely to encounter (and get infected) by parasites. Indeed, Amazon mollies were more likely to have at least one parasite when compared to Sailfin mollies. However, with no differences in parasite load, both species appear to display similar levels of quantitative parasite resistance—they are equally capable of resisting multiple infections. These findings are also consistent with previous work that showed no difference in parasite loads between Amazon and Sailfin mollies (Tobler and Schlupp 2005). Additionally, our study suggests that patterns of parasite prevalence and behavioral differences may be site‐dependent. As a result, environmental context may affect the strength of species‐level differences in behavior and parasite loads. Taken together, this study highlights how being asexual may not necessarily lead to higher parasite loads and that behavior may contribute to parasite exposure.

## Author Contributions

**Kirsten Sheehy:** conceptualization (lead), data curation (lead), formal analysis (lead), funding acquisition (lead), investigation (equal), methodology (equal), resources (equal), supervision (lead), validation (lead), visualization (lead), writing – original draft (lead), writing – review and editing (lead). **Jonathan Aguiñaga:** conceptualization (supporting), data curation (equal), formal analysis (supporting), investigation (equal), methodology (supporting), writing – original draft (supporting), writing – review and editing (supporting). **Mary Janecka:** investigation (equal), methodology (equal), writing – review and editing (supporting). **Jessica Stephenson:** investigation (equal), methodology (equal), resources (equal), writing – review and editing (supporting). **Kate L. Laskowski:** conceptualization (equal), formal analysis (equal), methodology (supporting), resources (equal), supervision (equal), writing – original draft (supporting), writing – review and editing (equal).

## Funding

We would like to disclose support for the research of this work from the Richard G. Coss Wildlife Research Award (3‐ANBCOSS).

## Conflicts of Interest

The authors declare no conflicts of interest.

## Data Availability

The data that support the findings of this study are openly available in Dryad at https://doi.org/10.5061/dryad.jsxksn0r9.
